# Randomized controlled trial to evaluate an app-based multimodal digital intervention for people with type 2 diabetes in comparison to a placebo app

**DOI:** 10.3389/fdgth.2025.1644612

**Published:** 2025-12-11

**Authors:** Lena Roth, Maxi Pia Bretschneider, Peter E. H. Schwarz

**Affiliations:** 1 Department for Prevention and Care of Diabetes, Department of Medicine III, Faculty of Medicine Carl Gustav Carus, Technical University Dresden, Dresden, Germany; 2 Paul Langerhans Institute Dresden, Faculty of Medicine, Technical University Dresden, Dresden, Germany

**Keywords:** HbA1c reduction, digital health intervention, diabetes type 2 (T2D), DiGA, diabetes self-management, well-being, lifestyle intervention

## Abstract

**Introduction:**

This multi-center, parallel-group randomized controlled trial evaluated the app-based intervention *mebix*, developed by Vision2b GmbH in Germany, for people with type 2 diabetes compared to a placebo app.

**Method:**

A total of 153 participants were randomized in a 1:1 ratio to either intervention or control group, with allocation concealment ensured by a minimization procedure.

**Results:**

After six months, participants using *mebix* achieved a statistically significant reduction in HbA1c levels by 0.82 percentage points (95% confidence interval: −1.20, −0.48, *p* = 0.003). This reduction was greater than in the control group (mean difference: 0.24 percentage points, 95% confidence interval: −0.44, 0.09). *mebix* users further experienced greater weight loss, lower diabetes-related distress, and reduced depression severity. Adherence to the app was high, with more than 75% of participants using *mebix* throughout the study period.

**Conclusion:**

These findings indicate that the digital approach can meaningfully improve both glycemic control and psychological well-being in people with type 2 diabetes, supporting its potential integration into routine care.

**Clinical Trial Registration:**

https://www.evamebix.de, identifier DRKS00025719, DRKS00032395.

## 1 Introduction

An estimated 589 million people worldwide, aged 20 to 79, are currently living with diabetes mellitus, with approximately 90% of these cases being type 2 diabetes mellitus (T2D) (1). In Germany, the prevalence of diabetes is estimated to range between 7.2% and 9.9%, comparable to rates observed in other European countries (1, 2). The prevalence of T2D increases with age, reaching an estimated 30% among German adults aged 75–79 (2). Among adults of age 60–99, Germany ranks among the top 10 countries worldwide in terms of the percentage of the population affected by T2D (1). Recent increases in both prevalence and incidence are expected to continue (3).

T2D is a metabolic disease characterized by chronically elevated blood glucose levels, caused by impaired insulin production and insulin resistance (4, 5). The diagnosis is based either on elevated levels of glycated hemoglobin (HbA1c ≥6.5% or ≥48 mmoL/moL), or plasma glucose [fasting plasma glucose: ≥126 mg/dL or ≥7.0 mmoL/L; 2 h glucose during an oral glucose tolerance test (OGTT): ≥200 mg/dL or ≥11.1 mmoL/L] (4). T2D is strongly linked to overweight and obesity, as well as elevated blood pressure and blood lipids (6, 7). These conditions increase the risk of cardiovascular complications, making people with T2D more vulnerable to strokes, heart failure, and myocardial infarction (1, 6, 7). In addition, T2D is associated with micro- and macrovascular complications, leading to comorbidities such as diabetic retinopathy and foot ulcers (1).

To reduce the risk of developing comorbidities, current guidelines for the management of T2D stress the importance of aiming to lower HbA1c levels. While treatment goals typically aim at a reduction of the HbA1c below a certain target usually defined between 7%–7.5%, they should be adapted based on the duration and severity of the disease (8–10). To improve glycemic control, therapy should consist of lifestyle modifications that support patients in the management of their blood glucose as well as their weight by increasing physical activity, improving dietary habits, and quitting smoking (7–10). When blood glucose control is no longer achievable with lifestyle modification and other blood glucose lowering therapies like metformin, sodium-glucose cotransporter-2 (SGLT2) inhibitors and glucagon-like peptide-1 receptor agonists (GLP-1), insulin treatment might be initiated (10).

Given its chronic nature, effective management also relies on regular blood glucose monitoring to enhance self-management and improve therapeutic outcomes (10, 11). To support people with T2D in their daily lives, diabetes technologies like (continuous) blood glucose meters, or comprehensive interventions in form of mobile applications, are becoming an integral part of treatment (10, 12, 13). People with T2D have a higher risk of developing depression and experiencing disease-related distress (14). Improving psychological well-being as well as self-management are central targets of behavioral interventions in T2D, especially given their mutual influence (15).

Since 2019, digital health applications (*Digitale Gesundheitsanwendungen*, DiGA) can be approved by the German Federal Institute for Drugs and Medical Devices (BfArM) if they are certified as medical devices and have demonstrated a positive effect on health care (16). Once listed in the official DiGA directory, these applications are eligible for prescription and are fully reimbursed by statutory health insurance providers. Current evidence shows the potential of such interventions to help patients improve their glycemic control, achieving HbA1c reductions of on average 0.8% (17).

The digital health application under investigation is *mebix* (developed by Vision2B GmbH, Germany), an app-based lifestyle intervention for people with T2D. For permanent listing as a DiGA, the current trial aims to provide the evidence for a positive health care impact according to § 139e of the German Social Code, Book V (Sozialgesetzbuch Fünftes Buch, SGB V) (16). The positive care effect is defined as improved glycemic control. Unlike previous DiGA trials, which so far relied on control groups that received usual care, this RCT represents the first trial within the diabetes DiGA framework to incorporate a placebo app.

The underlying hypotheses of the trial are 1) that participants in the intervention group (IG) achieve a reduction in HbA1c of at least 0.3 percentage points after six months (18), and 2) that the IG shows a superior reduction compared to the control group (CG) with respect to the HbA1c reduction at six months.

## 2 Methods

### 2.1 Study design and participants

The study is a multi-center, parallel-group, randomized-controlled trial. The research protocol was approved by the Ethics Committee of the Technical University Dresden (MDR ff-EK-421102023_2, Dresden, Germany). The trial was registered at the German Clinical Trials Register (DRKS00025719, DRKS00032395) and conducted in accordance with the declaration of Helsinki (19).

Participants were recruited between February and March 2024 via a study website (https://www.evamebix.de) or directly through the study centers. Interested participants that were recruited online filled in a first screening questionnaire with basic information (age, confirmed T2D diagnosis, availability of a smartphone, contact information) and chose a suitable study center. Afterwards patients were called by trained study personnel for a first screening based on defined inclusion and exclusion criteria (inclusion and exclusion criteria in Table 1) and to schedule a visit at the nearest study center. The target population included patients with suboptimal controlled T2D (HbA1c 7.5%–11%) likely to benefit from lifestyle interventions. Patients with HbA1c >11% or with severe psychological or physical impairments were excluded, because they might require different pharmacological and therapeutical approaches that take priority over a lifestyle-based digital intervention. Inclusion criteria also ensured participants could engage with the digital intervention, as assessed by study personnel during screening. At the baseline visit, eligible patients were informed about the study and gave written informed consent to participate in the study.

**Table 1:** Inclusion and exclusion criteria.

Inclusion	Exclusion
Patients ≥18 years old Patients with type 2 diabetes ICD-10: E11.-) who benefit from improved self-management Patients who have not used *mebix* in the past Patients willing to use *mebix* HbA1c at baseline ≥7.5% and ≤11.0% Participation in the DMP Diabetes for at least 6 months prior to the start of the study Patients with internet access and a smartphone compatible with *mebix* Digital literacy to use a smartphone appropriately and willingness to use it for their diabetes self-management Participants who are willing and able to comply with all scheduled visits, laboratory tests, lifestyle considerations and other study procedures Participants who are capable of providing a personal, signed informed consent form	Patients who use other apps for their diabetes management Patients who have used *mebix* Patients with impairments—including mental or psychological impairments—or conditions that, in the opinion of the investigator, would seriously jeopardize the integrity of the study Patients already using a continuous subcutaneous insulin infusion (CSII, “insulin pump”) Patients currently participating in a weight loss program Steroid therapy within the last three months (only topical or inhaled use is not an exclusion criterion as long as it is not more than five times per week) Uncontrolled blood pressure ≥200 mmHg at screening Current or planned pregnancy during the study or breastfeeding women Abuse of alcohol or illegal drugs in the last three months Patients with home care staff helping them with blood glucose monitoring and/or insulin adjustment Current participation in another study or examination Employees of Vision2B or another institution conducting the study or involved in the implementation / organization / coordination / funding of the study

Patients were included in the study between February 2 and September 29, 2024. The study was completed on March 4, 2025, with the final 6-month follow-up visit.

### 2.2 Randomization

Randomization (1:1) of eligible participants into IG or CG was conducted within the electronic case report form (eCRF; secuTrial) using an embedded variance minimization algorithm. The group assignment was triggered upon entry of the participant's baseline HbA1c value by the study personnel. This process allowed concealment of the allocation. The stratification was based on study center and baseline HbA1c level (<8%/≥8%) to ensure balanced distribution across these variables. Patients were randomized 1:1 into either IG or CG.

Although participants were blinded, study staff were not blinded due to their role in assisting with app setup and account activation.

### 2.3 Intervention

*mebix* is a CE-certified digital health application for patients with T2D [*International Classification of Diseases, Tenth Revision* (ICD-10) code E11.-]. The app was preliminarily approved from the BfArM by the time the study was conducted. This approval was based on the results of a separate pilot study that had demonstrated initial evidence of its benefit (20).

*mebix* aims at helping people with T2D to improve their self-management and lifestyle decisions to improve state of health and quality of life. It is a guideline-based therapy approach that can be used in addition to pharmacological or other therapies initiated by a physician. It is recommended to use *mebix* at least 6 months and up to 12 months. Within the app, users are guided and reminded through automated messages to set up their goals, track their physical activity and diet as well as physiological parameters. Information on diet and personalized nutrition, physical activity, mental well-being and social aspects of life with diabetes are provided through interactive educational multimedia content. A more detailed overview of the therapeutic approach was prescribed previously (20). For the control group, a placebo version of the app was used that was based on the original app but without the educational content; it only allowed tracking of certain aspects (e.g., nutrition, physical activity).

### 2.4 Experimental procedure

After the inclusion procedure (screening, randomization) at the assigned study center, the baseline measurements were assessed by the study personnel. Furthermore, patients received a tablet in order to complete patient-reported outcome measures (PROMs). They accessed the online questionnaires through personalized links provided via the secuTrial system.

Afterwards, participants received access either to the *mebix* app or the placebo app. After study completion, the CG was offered to also use *mebix* for 6 months. Participants received a compensation of €200-Amazon voucher for their study visits.

After the initial study visit for the baseline assessment, participants visited the study centers again after 3 and 6 months to conduct the follow-up visits. After the 6-months visit, patients were asked to participate in the extension of this trial, that is currently ongoing and aims at assessing the long-term effects of *mebix*. This extension of the study consists of an additional assessment at the study site 12 months after the original baseline visit.

### 2.5 Outcomes

The primary endpoint of interest is the change in HbA1c levels from baseline (T0) to 6 months. HbA1c levels were measured at the study centers by trained study personnel, following standardized procedures.

Secondary endpoints of interest included the change in body weight (in %) and diabetes-related distress from baseline to 6 months. Body weight was measured during the study visits by study personnel. As a patient-reported outcome, the diabetes-related distress was assessed via online questionnaires using the validated Problem Areas in Diabetes (PAID) scale (21, 22). It consists of 20 questions, each answered on a scale from 0 (not a problem) to 4 (serious problem), indicating the extent to which the items are currently a problem in the patient's life. The final score was calculated by adding up the answers over all questions and then multiplying them by 1.25. This yielded a total score of 100 with results above 40 indicating severe diabetes distress (23).

Further exploratory endpoints included self-efficacy assessed with the Short Scale for Measuring General Self-efficacy Beliefs (German: Allgemeine Selbstwirksamkeits Kurzskala) (24), quality of life assessed with the EQ-5D-5L (25), well-being assessed with the WHO-5 Well-Being Index (26, 27), empowerment, assessed with the Diabetes Empowerment Scale (DES-SF) (28), self-management, assessed with the Summary of Diabetes Self-Care Activities (SDSCA) (29) or depression severity assessed with the Patient Health Questionnaire-9 (PHQ-9) (30, 31).

#### 2.5.1 Sample size calculation

The sample size calculation was performed with the program G* power (32). Based on a previous umbrella review (33), an effect size of 0.5 was assumed. Supposing further an error probability of 0.05 and a power of 0.8; the sample size per group is 51. Considering a dropout of 20%, the total sample size is 132 patients.

### 2.6 Statistical analyses

Statistical analyses were performed using R version 4.3.3 within Rstudio version 2024.02.29. Baseline characteristics for all endpoints are presented using descriptive statistics.

The primary confirmatory analysis was based on the intention-to-treat (ITT) approach, including all randomized participants regardless of protocol compliance. To address confounding from major changes in antidiabetic medication, a modified ITT approach was applied for the primary endpoint whereby outcome data were censored from the time of medication adjustment onward. This allowed for full inclusion of participants while limiting bias introduced by pharmacological effects unrelated to the intervention.

Missing data were handled with multiple imputations (*n* = 100) using the R function *rbmi* version 1.4.1 and the implemented “copy increments to reference” (CIR) method, with the control group defined as the reference group (34–36). This method assumes that participants in the IG benefited from the treatment up to the point of dropout. After dropping out, their outcomes are assumed to follow the same trajectory as those in the CG (37). As a result, monotone missing values in the IG were imputed accordingly, while interim missing data were estimated based on the IG's trajectory. For the CG, monotone and interim missing data were imputed based on parameters derived from the CG itself. All of the covariates that were part of the regression model were also used as covariates for imputation.

Differences in endpoints at 3 and 6 months were calculated after imputing the raw values at each time point. Linear mixed models were fitted to each of the resulting imputed data sets and pooled using a multivariate Wald test using the R package *mitml* version 0.4–5 (38, 39). The independent variables of these models were the within-group factor *time* (3 and 6 months) and the between-group factor *group* (IG and CG); included in the model as interacting factors.

To control for potential confounding variables, the baseline HbA1c (included as an interacting factor with time in the model for the primary endpoint), the study center and gender were included. Moreover, a random intercept for each participant was included. Regarding secondary and exploratory endpoints, the model further comprised the baseline value of the respective endpoint as an additional confounding variable.

The results, i.e., mean (M), standard deviation (SD) and 95% confidence intervals (CI) are thus adjusted for all included covariates.

Finally, hypotheses were tested using *a priori* defined contrasts or one-sided one-sample t-tests, using the R package *emmeans* version 1.10.1. Due to multiple testing, adjustments for multiplicity were performed using the Benjamini-Hochberg false discovery rate (FDR) correction, applied separately to hypotheses regarding the primary and secondary endpoints. As an additional effect size measure, Cohens' *d* and the corresponding 95% CI were reported based on the *t* values from contrasts and one-sample *t* tests.

A per-protocol (PP) approach was used as a second analysis. Participants who did not comply with the study protocol were excluded, i.e., participants who dropped out or no longer fulfilled inclusion criteria as well as participants in the IG who did not use *mebix* at all.

## 3 Results

### 3.1 Participant characteristics and participant flow

Between the 2nd February 2024 and the 29th September 2024, 153 patients were included in the study and randomized either into the IG or the CG.

Overall, 60.1% of participants were male. At baseline the average age was 59.1 years ±9.9 (M ± SD) and the average baseline HbA1c value 8.56 percentage points ±0.96 (M ± SD). Tables 2, 3 give an overview of the baseline demographics and the endpoint characteristics, showing that the groups were comparable. For the secondary and exploratory endpoints, baseline data is not available for the whole randomized sample because one patient of the CG did not fill in any baseline questionnaire. Additional missing values are attributable to patients either omitting responses to individual questions or failing to complete entire questionnaires.

**Table 2:** Demographic baseline characteristics of patients by group (ITT sample).

Variable	CG	IG	Overall
	(*N* = 77)	(*N* = 76)	(*N* = 153)
Age			
Mean (SD)	59.7 (8.52)	58.5 (11.2)	59.1 (9.92)
[Min, Max]	60.0 [29.0, 78.0]	58.5 [16.0, 81.0]	59.0 [16.0, 81.0]
Gender			
Female	32 (41.6%)	29 (38.2%)	61 (39.9%)
Male	45 (58.4%)	47 (61.8%)	92 (60.1%)
HbA1c (in %)			
Mean (SD)	8.61 (0.993)	8.51 (0.930)	8.56 (0.960)
Median [Min, Max]	8.30 [7.40, 10.9]	8.25 [7.40, 11.6]	8.30 [7.40, 11.6]
HbA1c Category			
<8%	25 (32.5%)	25 (32.9%)	50 (32.7%)
≥8%	52 (67.5%)	51 (67.1%)	103 (67.3%)
Years since diagnosis			
Mean (SD)	11.5 (8.66)	12.2 (7.46)	11.9 (8.07)
Median [Min, Max]	10.0 [0, 39.0]	10.0 [0, 31.0]	10.0 [0, 39.0]
Missing	3 (3.9%)	4 (5.3%)	7 (4.6%)
BMI (kg/m^2^)			
Mean (SD)	33.4 (5.97)	33.3 (5.65)	33.4 (5.80)
Median [Min, Max]	32.9 [24.1, 57.1]	33.7 [19.5, 49.4]	33.1 [19.5, 57.1]
BMI Category			
Grad I	28 (36.4%)	19 (25.0%)	47 (30.7%)
Grad II	19 (24.7%)	24 (31.6%)	43 (28.1%)
Grad III	7 (9.1%)	7 (9.2%)	14 (9.2%)
<25 kg/m^2^	23 (29.9%)	26 (34.2%)	49 (32.0%)
Weight (in kg)			
Mean (SD)	101 (16.6)	100 (20.7)	100 (18.7)
Median [Min, Max]	98.0 [69.4, 148]	98.4 [47.5, 171]	98.0 [47.5, 171]
Waist Circumference (in cm)			
Mean (SD)	117 (13.1)	116 (15.4)	116 (14.2)
Median [Min, Max]	117 [79.0, 158]	115 [58.0, 157]	115 [58.0, 158]
Missing	9 (11.7%)	12 (15.8%)	21 (13.7%)
Smoker			
Yes	22 (28.6%)	13 (17.1%)	35 (22.9%)
No	47 (61.0%)	59 (77.6%)	106 (69.3%)
Missing	8 (10.4%)	4 (5.3%)	12 (7.8%)

CG, control group; IG, intervention group; ITT, intention-to-treat; SD, standard deviation; min, minimum; max, maximum

**Table 3:** Baseline values of patient-reported outcome measures by group (ITT sample).

Variable	CG	IG	Overall
	(*N* = 76)	(*N* = 76)	(*N* = 152)
Well-being (WHO-5)			
Mean (SD)	14.0 (5.71)	13.8 (5.60)	13.9 (5.63)
Median [Min, Max]	15.0 [2.00, 25.0]	14.0 [0, 24.0]	15.0 [0, 25.0]
Impaired well-being	27 (35.5%)	29 (38.2%)	56 (36.8%)
Self-efficacy (ASKU)			
Mean (SD)	4.00 (0.564)	3.90 (0.968)	3.95 (0.791)
Median [Min, Max]	4.00 [2.33, 5.00]	4.00 [0, 5.00]	4.00 [0, 5.00]
Diabetes-empowerment (DES-SF)			
Mean (SD)	3.39 (0.510)	3.39 (0.586)	3.39 (0.548)
Median [Min, Max]	3.46 [2.21, 4.54]	3.45 [0.107, 4.21]	3.46 [0.107, 4.54]
Diabetes-related distress (PAID)			
Mean (SD)	31.3 (17.4)	33.2 (20.1)	32.2 (18.8)
Median [Min, Max]	28.8 [1.25, 72.5]	30.6 [1.25, 78.8]	29.4 [1.25, 78.8]
Severe distress	22 (28.9%)	30 (39.5%)	52 (34.2%)
Patient health (PHQ-9)			
Mean (SD)	7.47 (4.59)	8.08 (5.83)	7.78 (5.24)
Median [Min, Max]	7.00 [0, 17.0]	7.00 [0, 24.0]	7.00 [0, 24.0]
Mild symptoms	22 (28.9%)	26 (34.2%)	48 (31.6%)
Moderate symptoms	20 (26.3%)	13 (17.1%)	33 (21.7%)
Moderate severe symptoms	6 (7.9%)	7 (9.2%)	13 (8.6%)
Severe symptoms	0 (0%)	4 (5.3%)	4 (2.6%)
Physical quality of life			
Mean (SD)	54.4 (4.25)	53.2 (3.68)	53.8 (4.01)
Median [Min, Max]	56.6 [46.4, 61.9]	52.0 [46.4, 61.9]	55.7 [46.4, 61.9]
Psychological Quality of Life			
Mean (SD)	58.3 (4.95)	58.3 (4.48)	58.3 (4.70)
Median [Min, Max]	60.8 [48.2, 63.9]	60.8 [48.2, 63.9]	60.8 [48.2, 63.9]

ASKU, Short Scale for Measuring General Self-efficacy Beliefs (German: Allgemeine Selbstwirksamkeits Kurzskala); CG, control group; IG, intervention group; SD, standard deviation; ITT, intention-to-treat; min, minimum; max, maximum.

**Figure 1:**
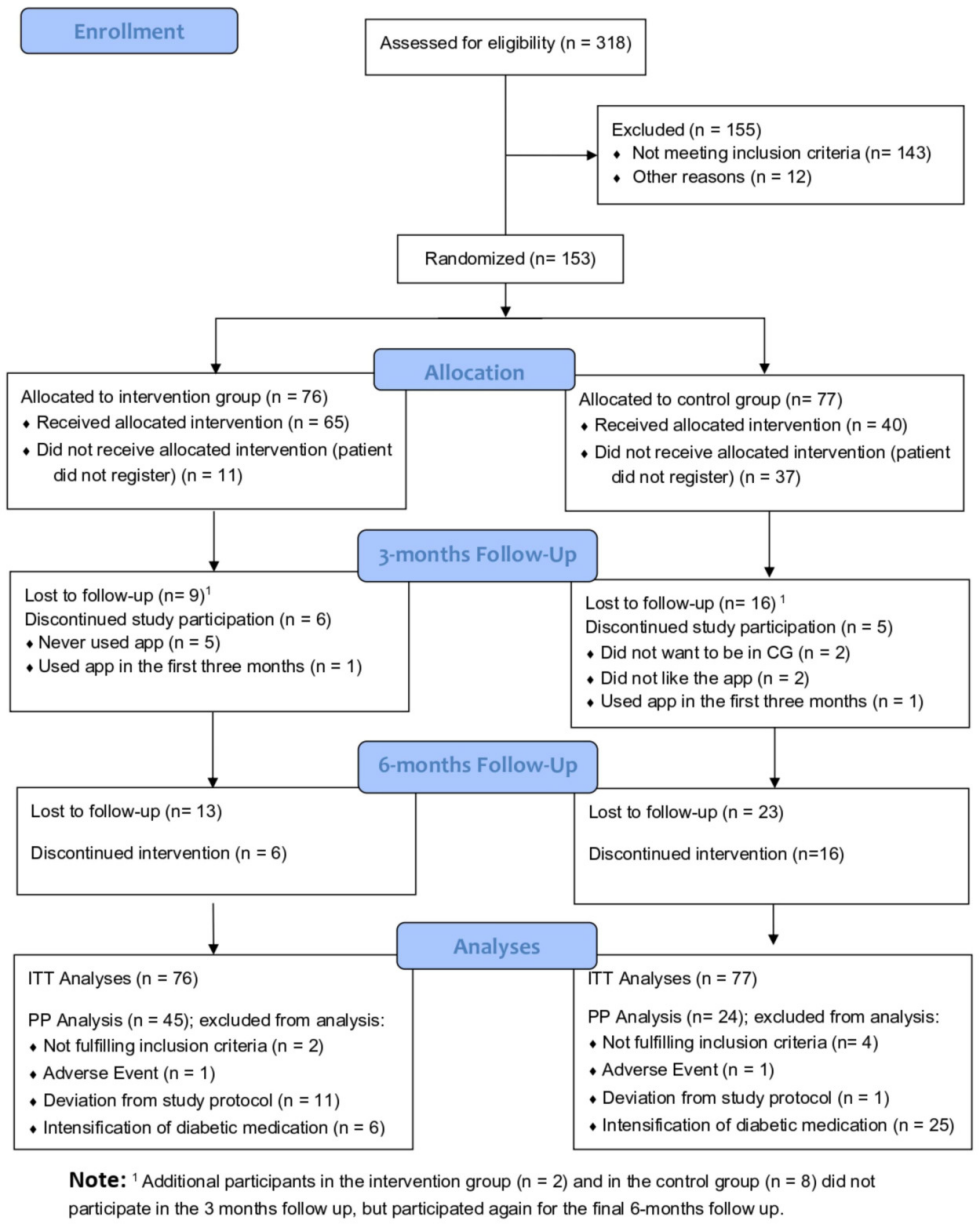
Patient flow chart. Note: CG, control group; IG, intervention group; ITT, intention-to-treat; PP, per-protocol.

Figure 1 shows the participant flow, including reasons for exclusion from the PP analysis (some participants fell into more than one category for exclusion, see Supplementary Information S1). The most common reason to exclude patients was dropout/loss to follow-up, mostly occurring in the first three months of the study. When comparing the ITT and the PP sample, patients in the PP are more often female and tend to have a lower baseline weight (and BMI) (see Supplementary Information S2).

### 3.2 Primary end point

Based on the ITT analysis, participants in the IG reduced their HbA1c levels an average of −0.80 percentage points (95% CI: [−1.14, −0.46]) after 6 months. This confirmed the hypothesis that *mebix* leads to a relevant reduction in HbA1c levels of at least 0.3%, with a moderate to large effect size [*t*(136) = −4.632, *p*_FDR_ = 0.004, *d* = −0.79 [−1.14, −0.44]]. Comparable results were observed in the PP analysis, supporting the robustness of the findings [mean HbA1c reduction: −0.89 percentage points, 95% CI: [−1.17, −0.61], *t*(93) = −6.327, *p*_FDR_ < 0.001, *d* = −1.31 [−1.76, −0.86]].

**Figure 2:**
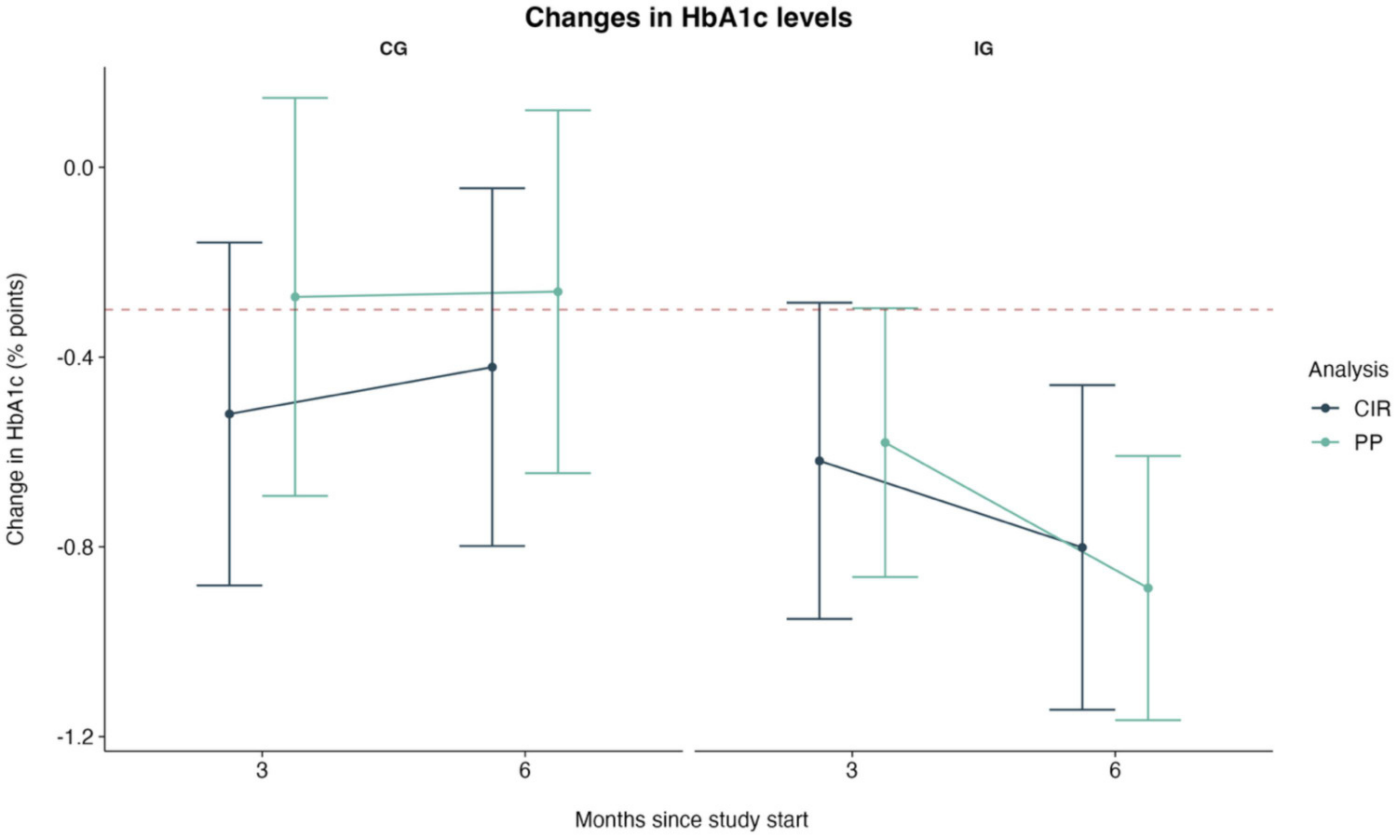
Adjusted mean changes in HbA1c levels (in percentage points) and 95% confidence intervals by analysis and group. The red dotted line shows the clinically relevant reduction in HbA1c levels of at least 0.3%. Note: CG, control group; CIR, copy increments to reference; IG, intervention group; PP, Per-Protocol.

While the ANCOVA in the ITT sample did not yield significant main or interaction effects for *time* and *group*, the factor group was significant in the PP analysis (Supplementary Information S4). Across analyses, *baseline HbA1c* was a significant covariate, indicating greater reductions among participants with higher baseline HbA1c levels. Accordingly, the ITT analysis showed that HbA1c reductions were on average 0.38 percentage points greater in the IG than the CG [95% CI: [−0.75, −0.01]; *t*(128) = −2.010, *p*_FDR_ = 0.047, *d* = −0.35 [−0.70, 0.00]]. The PP analysis confirmed this difference [mean difference: −0.62 [−1.10, −0,15], *t*(92) = −2.611, *p*_FDR_ = 0.011, *d* = −0.54 [−0.96, −0.13]]. Reductions continued from 3 to 6 months in the CIG, while the CG stagnated or even slightly increased (Figure 2). Overall, significantly more patients in the IG achieved treatment goals, including a relevant HbA1c reduction of at least 0.3% or levels below 7% (Supplementary Information S3).

Overall, both the ITT and the PP analyses support the effectiveness of mebix regarding the glycemic control of people with T2D.

### 3.3 Secondary endpoints

In the ITT-based ANCOVA for weight change, the main factor *time* was significant, indicating that weight changed over the study period (Supplementary Information S4). For diabetes-related distress, only the baseline level of *diabetes-related distress* was a significant covariate, suggesting that observed changes were largely dependent on participants' initial distress level (Supplementary Information S4). Comparable results were obtained in the PP analyses, with the additional finding that the main factor *group* reached significance for diabetes-related distress, indicating favorable results in the IG compared to the CG (Supplementary Information S4).

The one-sample t-tests defined *a priori* confirmed that participants using *mebix* showed significant improvements in both secondary endpoints after 6 months, regardless of analysis population. While these improvements were greater in the IG than the CG, they were not significant (see Table 4). Taken together, the consistency across ITT and PP analyses supports the robustness of these findings.

**Table 4:** Results of the hypotheses testing of the secondary endpoints weight change (in %) and diabetes-related distress after 6 months for the intention-to-treat and the per-protocol sample.

Hypothesis	Analysis	Inference				
		Estimate (95% CI)	*t*-ratio	df	pFDR	*d* (95% CI)
Weight (in %)						
IG: Baseline >6 months	CIR	−1.6 [−2.85, −0.35]	−2.524	223	0.012*	−0.34 [−0.6, −0.07]
IG < CG	CIR	−0.28 [−2.05, 1.49]	−0.315	225	0.753	−0.04 [−0.3, 0.22]
IG: Baseline >6 months	PP	−1.11 [−3.07, 0.86]	−1.124	72	<.0001***	−0.27 [−0.73, 0.2]
IG < CG	PP	0.83 [−1.78, 3.43]	0.632	83	0.176	0.14 [−0.29, 0.57]
Diabetes-related distress						
IG: Baseline >6 months	CIR	−8.38 [−12.95, −3.81]	−3.620	185	0.001***	−0.53 [−0.83, −0.24]
IG < CG	CIR	−2.61 [−7.40, 2.19]	−1.071	229	0.380	−0.14 [−0.40, 0.12]
IG: Baseline >6 months	PP	−11.12 [−16.74, −5.51]	−3.947	75	<.0001***	−0.91 [−1.38, −0.43]
IG < CG	PP	−5.58 [−12.65, 1.48]	−1.570	86	0.176	−0.34 [−0.76, 0.09]

Significance codes: 0 “***” 0.001 “**” 0.01 “*” 0.05 “.” 0.1 “ ” 1.

CG, control group; CI, Confidence-Interval; CIR, copy increments to reference; *d*, Cohens' d; df, degrees of freedom; IG, intervention group; pFDR, *p*-value corrected by the false discovery rate; PP, Per-Protocol.

### 3.4 Exploratory endpoints

Further exploratory endpoints showed positive developments in the IG, with greater improvements than the CG for most outcomes. Detailed results are summarized in the Supplementary Information S5,S6. Apart from predefined exploratory endpoints, the majority of IG participants used *mebix* throughout the study period, indicating good adherence and acceptance of the intervention (see Figure 1).

## 4 Discussion

This study aimed to demonstrate a positive healthcare effect of *mebix* defined by a reduction of the HbA1c levels by at least 0.3 percentage points in patients with T2D over a 6-month period. This positive healthcare effect was demonstrated in the primary confirmatory mITT analysis and supported by the PP analysis.

According to the ITT analysis, participants using *mebix* achieved a significant and clinically relevant reduction in HbA1c levels, with an average decrease of 0.82 percentage points. The PP analysis also showed significant reductions in the IG after 6 months with a large effect size. These reductions are within or above the range reported in comparable RCTs evaluating DiGA-like interventions (17, 40).

Consistent with other studies on digital diabetes interventions, the greatest HbA1c reduction occurred within the first three months (17, 33). Contrary to earlier findings, the IG in this study continued to reduce their HbA1c levels beyond the initial three months, while the CG either stagnated or experienced increases in HbA1c levels. After 6-months the IG achieved significantly greater HbA1c reductions compared to the CG. This indicates that self-observation in terms of tracking (placebo app) is insufficient for achieving long-term behavioral change, and that educational content—as provided by the *mebix* app—is necessary to further improve HbA1c levels and sustain therapeutic outcomes.

Similar to previous trials that evaluated the efficacy of a DiGA for people with T2D, antidiabetic treatment during the study period was started or increased more often in the CG (*n* = 16) than in the IG (*n* = 6) (40). This shows that DiGA might contribute to stabilize HbA1c levels, thereby reducing the need for pharmacological escalation in patient with T2D.

Significant improvements in the secondary endpoints, diabetes-related distress and weight demonstrated positive health effects of *mebix* and are again comparable to another trial analyzing the efficacy of another T2D DiGA (40). While weight change showed a small effect size, the reduction in diabetes-related distress was medium to large, reflecting greater intervention benefits for this outcome. The modest weight change may be partly due to non-fasting measurements, with 61% of CG participants and 70% of IG participants weighed after consuming food or drinks. Changes in body composition, such as fat loss and increased muscle mass from enhanced physical activity promoted by *mebix*, may also explain this [comp (41)]. Indeed, the waist circumferences decreased in the IG but not in the CG. Future studies should employ methods such as bioelectrical impedance analysis to better quantify changes in body composition. PP analyses further support mental health benefits, showing reductions in diabetes-related distress and depression severity in the IG. Finally, the significant increase in empowerment among IG participants highlights the educational potential of *mebix* to enhance self-management and, ultimately, improving glycemic outcomes.

### 4.1 Strength and limitations

A key strength of the present study is the high adherence observed in the IG, also in comparison to other DiGA trials (41–43). Most participants (75%) used the *mebix* app during the whole study period. This high level of adherence is also reflected in the user experience ratings, particularly for *perspicuity, attractiveness* and *dependability* (Supplementary Information S6). In contrast, dropout in the control group (CG) was three times higher (*n* = 21; 27.3%), even without accounting for participants who never registered for the placebo app. This suggests lower acceptance and satisfaction with the CG intervention, leading to higher discontinuation.

This study further addresses a common criticism in the body of evidence of DiGA i.e., is the lack of blinding and an active control group. It is the first diabetes DiGA trial to implement a placebo app (44, 45). Unfortunately, the implementation of such an app has not proven to be straightforward. Several participants in the CG were aware of their treatment allocation and never used the app. It is likely that participants informed themselves about *mebix* and recognized that the placebo app lacked relevant components. This highlights the difficulty of maintaining blinding in digital intervention studies.

A potential limitation is the selection bias inherent in the study population. Participants were likely already interested in *mebix* and digital interventions for diabetes management which may limit generalizability to the broader population of patients with T2D. Nonetheless, the baseline characteristics of the study sample align with the average *mebix* user (46), suggesting that the findings are applicable to real-world users who are motivated to engage with digital interventions.

Another challenge in evaluating digital intervention for diabetes is the influence of medication. A continuous adaption of medication is in line with the development of the disease of T2D patients and reflects the standard of care (9). Future studies and analysis should consider methods to account for the complex interplay of diabetes medication and the studied intervention, for example adjusting outcomes with medical effect scores to more accurately isolate the intervention's effects (47).

Lastly, exploratory outcomes may overlap conceptually (e.g., self-efficacy and empowerment, self-management and well-being). However, since these endpoints were considered exploratory, no formal multicollinearity testing was performed, and results should be interpreted accordingly.

## 5 Conclusion

Overall, this study provides robust evidence supporting the effectiveness of *mebix*. The significant and clinically meaningful reduction in HbA1c levels in the IG demonstrated the positive healthcare effect of *mebix*. Compared to the CG using a placebo app, the intervention effects in the IG were more pronounced. In addition, *mebix* lead to significant improvements in patient-reported outcomes, including diabetes-related distress and depression severity, as well as enhanced self-management and empowerment. These findings highlight the multifaceted benefits of *mebix* in supporting both glycemic control and psychosocial well-being in people with T2D.

## Data Availability

The original contributions presented in the study are included in the article/Supplementary Material, further inquiries can be directed to the corresponding author.
