# Supplementary material for: Randomized controlled trial to evaluate an app-based multimodal digital intervention for people with type 2 diabetes in comparison to a placebo app
Source: Front Digit Health. 2025 Dec 11;7:1644612. doi: 10.3389/fdgth.2025.1644612 (PMC12738949; doi:10.3389/fdgth.2025.1644612)
Supplement: Supplementary file 1 [file Datasheet1.pdf]

# *Supplementary Material*

## 1) Reason for exclusion from the per-protocol sample

As shown in Figure 1 (main manuscript), 45 (59.2%) of IG and 24 (31.2%) of CG participants of the originally randomized participant are included in the PP analysis.

The most common reason to exclude patients was dropout / loss to follow up (IG = 13; CG = 23). All dropouts happened in the first three months of the study (IG = 6, CG = 5). Most cases of lost to follow up occurred in the first three months; in the nine out of 13 and in the CG 16 out of 23.

Further reasons to exclude patients from the PP analyse were HbA1c outside the inclusion range. In the CG[1] four participants and in the IG one participant had HbA1c values of 7.4% and therefore slightly below the defined inclusion range of 7.5%. Additionally, one participant in the IG had a HbA1c above the defined range of 11.6% and one participant in the IG severe health impairments compromising the study participation (n = 1).

While some participants of the IG who dropped out also never used *mebix* or were lost to follow up (n = 8), others stayed in the study (n = 3). The latter once were excluded from the PP analysis due to non-adherence to the study protocol.

Overall, adverse events (AEs) occurred once in the CG[2] and once in the IG during the study period. As a result, one more IG participants was excluded from the PP analysis due to the death of a family member. The initiation and intensification of anti-diabetic drugs at baseline and after 3 months was another strongly concomitant therapeutic decision that led to the exclusion of participants from the PP analysis (see Table 1). Lastly, one CG participants had the 6 months follow-up outside the time-window and therefore was excluded due to deviation from the study protocol.

**Table 1: Initiation and intensification of anti-diabetic drugs during the study period.**

| **Anti-diabetic drug class** | **Timepoint** | **IG** | | **CG** | |
|---|---|---|---|---|---|
| | | Start of new medication | Intensification | Start of new medication | Intensification |
| Insulin | At baseline | 0 | 0 | 1 | 0 |
| GLP-1-RA | At baseline | 0 | 0 | 5 | 0 |
| SGLT-1 | At baseline | 0 | 0 | 2 | 0 |
| Metformin | At baseline | 0 | 0 | 4 | 0 |

[1] One of these participants was also a dropout.

[2] The participant was also a drop out due to alcoholism and the initiation of therapy with antidepressants.

| **Anti-diabetic drug class** | **Timepoint** | **IG** | | **CG** | |
|---|---|---|---|---|---|
| | | Start of new medication | Intensification | Start of new medication | Intensification |
| DPP-4 | At baseline | 0 | 0 | 0 | 0 |
| Insulin | After 3 months | 0 | 0 | 5 | 0 |
| GLP-1-RA | After 3 months | 4 | 0 | 3 | 4 |
| SGLT-1 | After 3 months | 1 | 1 | 6 | 1 |
| Metformin | After 3 months | 0 | 1 | 1 | 0 |
| DPP-4 | After 3 months | 2 | 0 | 1 | 0 |
| Insulin | After 6 months | 0 | 0 | 0 | 0 |
| GLP-1-RA | After 6 months | 0 | 0 | 0 | 2 |
| SGLT-1 | After 6 months | 0 | 0 | 0 | 0 |
| Metformin | After 6 months | 0 | 0 | 0 | 0 |
| DPP-4 | After 6 months | 0 | 1 | 0 | 1 |
| Gesamt | | 7 | 3 | 28 | 8 |

## 2) Endpoint and demographic characteristics of the per-protocol and intention-to-treat sample at baseline

**Supplementary Table 1: General baseline and main and secondary endpoint characteristics of patients by group and sample.**

| | CG | | IG | | Overall | |
|---|---|---|---|---|---|---|
| | ITT (N=77) | PP (N=32) | ITT (N=76) | PP (N = 45) | ITT (N=153) | PP (N=77) |
| **Age** | | | | | | |
| *Mean (SD)* | 59.7 (8.52) | 61.8 (8.58) | 58.5 (11.2) | 60.8 (9.06) | 59.1 (9.92) | 61.1 (8.85) |
| *Median [Min, Max]* | 60.0 [29.0, 78.0] | 64.5 [46.0, 75.0] | 58.5 [16.0, 81.0] | 62.0 [43.0, 78.0] | 59.0 [16.0, 81.0] | 63.0 [43.0, 78.0] |
| **Gender** | | | | | | |
| *female* | 32 (41.6%) | 11 (45.8%) | 29 (38.2%) | 22 (48.9%) | 61 (39.9%) | 33 (47.8%) |
| *male* | 45 (58.4%) | 13 (54.2%) | 47 (61.8%) | 23 (51.1%) | 92 (60.1%) | 36 (52.2%) |
| **HbA1c (in %)** | | | | | | |
| *Mean (SD)* | 8.61 (0.993) | 8.21 (0.767) | 8.51 (0.930) | 8.42 (0.798) | 8.56 (0.960) | 8.34 (0.788) |
| *Median [Min, Max]* | 8.30 [7.40, 10.9] | 8.00 [7.50, 10.8] | 8.25 [7.40, 11.6] | 8.20 [7.50, 10.4] | 8.30 [7.40, 11.6] | 8.10 [7.50, 10.8] |

| | | | | | | |
|---|---|---|---|---|---|---|
| **HbA1c Category** | | | | | | |
| *< 8%* | 25 (32.5%) | 11 (45.8%) | 25 (32.9%) | 13 (28.9%) | 50 (32.7%) | 24 (34.8%) |
| *≥ 8%* | 52 (67.5%) | 13 (54.2%) | 51 (67.1%) | 32 (71.1%) | 103 (67.3%) | 45 (65.2%) |
| **Years since diagnosis** | | | | | | |
| *Mean (SD)* | 11.5 (8.66) | 14.9 (10.0) | 12.2 (7.46) | 12.4 (7.23) | 11.9 (8.07) | 13.3 (8.33) |
| *Median [Min, Max]* | 10.0 [0, 39.0] | 12.0 [1.00, 34.0] | 10.0 [0, 31.0] | 10.5 [0, 27.0] | 10.0 [0, 39.0] | 11.0 [0, 34.0] |
| *Missing* | 3 (3.9%) | 1 (4.2%) | 4 (5.3%) | 3 (6.7%) | 7 (4.6%) | 4 (5.8%) |
| **BMI (kg/m$^2$)** | | | | | | |
| *Mean (SD)* | 33.4 (5.97) | 32.4 (4.39) | 33.3 (5.65) | 32.7 (5.43) | 33.4 (5.80) | 32.6 (5.06) |
| *Median [Min, Max]* | 32.9 [24.1, 57.1] | 31.5 [26.0, 40.9] | 33.7 [19.5, 49.4] | 32.0 [19.5, 49.4] | 33.1 [19.5, 57.1] | 32.0 [19.5, 49.4] |
| **BMI Category** | | | | | | |
| *Grad I* | 28 (36.4%) | 11 (45.8%) | 19 (25.0%) | 16 (35.6%) | 47 (30.7%) | 27 (39.1%) |
| *Grad II* | 19 (24.7%) | 5 (20.8%) | 24 (31.6%) | 11 (24.4%) | 43 (28.1%) | 16 (23.2%) |
| *Grad III* | 7 (9.1%) | 1 (4.2%) | 7 (9.2%) | 3 (6.7%) | 14 (9.2%) | 4 (5.8%) |

| | | | | | | |
|---|---|---|---|---|---|---|
| *< 25 kg / m²* | 23 (29.9%) | 7 (29.2%) | 26 (34.2%) | 15 (33.3%) | 49 (32.0%) | 22 (31.9%) |
| **Weight (in kg)** | | | | | | |
| *Mean (SD)* | 101 (16.6) | 97.2 (16.2) | 100 (20.7) | 96.9 (20.1) | 100 (18.7) | 97.0 (18.7) |
| *Median [Min, Max]* | 98.0 [69.4, 148] | 96.8 [69.4, 142] | 98.4 [47.5, 171] | 98.0 [47.5, 171] | 98.0 [47.5, 171] | 97.0 [47.5, 171] |
| **Diabetes-related distress (PAID)** | | | | | | |
| *Mean (SD)* | 31.3 (17.4) | 27.7 (16.1) | 33.2 (20.1) | 33.2 (19.2) | 32.2 (18.8) | 31.3 (18.3) |
| *Median [Min, Max]* | 28.8 [1.25, 72.5] | 29.4 [2.50, 66.25] | 30.6 [1.25, 78.8] | 28.8 [1.25, 78.8] | 29.4 [1.25, 78.8] | 28.8 [1.25, 78.8] |
| **Center** | | | | | | |
| *Berlin* | 17 (22.1%) | 4 (16.7%) | 15 (19.7%) | 8 (17.8%) | 32 (20.9%) | 12 (17.4%) |
| *Dresden* | 14 (18.2%) | 9 (37.5%) | 15 (19.7%) | 13 (28.9%) | 29 (19.0%) | 22 (31.9%) |
| *Duisburg* | 10 (13.0%) | 0 (0%) | 11 (14.5%) | 5 (11.1%) | 21 (13.7%) | 5 (7.2%) |
| *Essen* | 32 (41.6%) | 8 (33.3%) | 31 (40.8%) | 17 (37.8%) | 63 (41.2%) | 25 (36.2%) |
| *Münster* | 3 (3.9%) | 2 (8.3%) | 3 (3.9%) | 1 (2.2%) | 6 (3.9%) | 3 (4.3%) |

| | | | | | | |
|---|---|---|---|---|---|---|
| *Osnabrück* | 1 (1.3%) | 1 (4.2%) | 1 (1.3%) | 1 (2.2%) | 2 (1.3%) | 2 (2.9%) |

*Abbreviations: CG, control group; IG; intervention group; ITT, intention-to-treat; PAID, Problem Areas in Diabetes Scale; PP, per-protocol.*

## 3) Number of patients achieving treatment goals per group

**Supplementary Table 2: Statistics for the achievement of treatment goals by group (intention-to-treat sample).**

| | Estimate and 95% confidence intervals | p-value |
|---|---|---|
| **< 6.5%** | | |
| Odds Ratio | 1.56 [0.35; 7.85] | 0.499 |
| Risk Ratio | 1.22 [ 0.71; 2.09] | 0.499 |
| Risk Difference | 0.03 [-0.05; 0.11] | 0.499 |
| **< 7%** | | |
| Odds Ratio | 2.47 [0.93; 7.12] | 0.043* |
| Risk Ratio | 1.48 [1.06; 2.05] | 0.043* |
| Risk Difference | 0.12 [0.00; 0.24] | 0.043* |
| **Responder** | | |
| Odds Ratio | 2.19 [1.09; 4.48] | 0.015* |
| Risk Ratio | 1.51 [1.06; 2.15] | 0.015* |
| Risk Difference | 0.19 [0.04; 0.34] | 0.015* |

*Significance Codes: 0 '***' 0.001 '**' 0.01 '*' 0.05 '.' 0.1 ' ' 1*

4) **Results of the ANCOVA for the primary and secondary endpoints based on the intention-to-treat and per-protocol samples**

**Supplementary Table 3: Results of the ANCOVA for the primary endpoint based on the per-protocol and intention-to-treat sample by analysis.**

| Analysis | Effect | *df* | *F-value* | *p-value* |
|---|---|---|---|---|
| | | **HbA1c change (in percentage points)** | | |
| **CIR** | time | 1, 212.87 | 1.88 | 0.171 |
| **CIR** | group | 1, 227.26 | 0.28 | 0.599 |
| **CIR** | baseline HbA1c | 1, 150.5 | 4.29 | 0.04* |
| **CIR** | gender | 1, 218.18 | 1.51 | 0.221 |
| **CIR** | center | 5, 283.8 | 0.07 | 0.996 |
| **CIR** | group * time | 1, 189.82 | 2.32 | 0.129 |
| **CIR** | baseline HbA1c * time | 1, 199.07 | 1.52 | 0.22 |
| **PP** | time | 1, 59.98 | 0.84 | 0.363 |
| **PP** | group | 1, 62.85 | 4.57 | 0.036* |
| **PP** | baseline HbA1c | 1, 63.1 | 4.33 | 0.042* |
| **PP** | gender | 1, 59.6 | 2.00 | 0.162 |
| **PP** | center | 1, 61.45 | 0.13 | 0.721 |
| **PP** | group * time | 1, 59.76 | 1.77 | 0.188 |
| **PP** | baseline HbA1c * time | 1, 60.3 | 1.07 | 0.306 |
| | | **Weight change (in %)** | | |
| **CIR** | time | 1, 166 | 5.11 | 0.025* |

| | | | | |
|---|---|---|---|---|
| **CIR** | group | 1, 264 | 0.86 | 0.354 |
| **CIR** | baseline BMI | 1, 256 | 3.10 | 0.079 |
| **CIR** | baseline HbA1c | 1, 221 | 2.91 | 0.090 |
| **CIR** | gender | 1, 237 | 0.23 | 0.629 |
| **CIR** | center | 5, 282 | 1.83 | 0.108 |
| **PP** | time | 1, 68 | 2.87 | 0.095 |
| **PP** | group | 1, 69 | 0.13 | 0.716 |
| **PP** | baseline BMI | 1, 68 | 1.22 | 0.274 |
| **PP** | baseline HbA1c | 1, 71 | 0.82 | 0.368 |
| **PP** | gender | 1, 69 | 0.25 | 0.615 |
| **PP** | center | 5, 68 | 1.01 | 0.42 |
| **PP** | group * time | 1, 69 | 0.36 | 0.548 |
| | | **Diabetes-related distress (PAID)** | | |
| **CIR** | time | 1, 178 | 1.83 | 0.178 |
| **CIR** | group | 1, 240 | 0.47 | 0.492 |
| **CIR** | baseline PAID | 1, 174 | 0.63 | 0.428 |
| **CIR** | baseline HbA1c | 1, 227 | 40.43 | < .0001*** |
| **CIR** | gender | 1, 198 | 0.03 | 0.853 |
| **CIR** | center | 5, 277 | 0.12 | 0.987 |
| **PP** | time | 1, 60 | 0.49 | 0.488 |
| **PP** | group | 1, 66 | 6.41 | 0.014* |

| | | | | |
|---|---|---|---|---|
| **PP** | baseline PAID | 1, 62 | 17.88 | < .0001*** |
| **PP** | baseline HbA1c | 1, 63 | 2.51 | 0.118 |
| **PP** | gender | 1, 64 | 0.00 | 0.95 |
| **PP** | center | 5, 65 | 0.49 | 0.784 |

*Significance Codes: 0 '***' 0.001 '**' 0.01 '*' 0.05 '.' 0.1 ' ' 1*

*Abbreviations: CIR, copy increments to reference; df, degrees of freedom; LOCF, Last observation carried forward; PP, Per-Protocol*

5) **Results of additional exploratory Endpoints**

After 6 months of using *mebix*, the participants in the IG show improvements over almost all exploratory endpoints, i.e. increases in well-being, (physical and psychological) quality of life and self-management as well as a decrease in depression severity. While the CG also showed improvements, they were less pronounced, and the psychological quality of life decreased (Supplementary Table 4). The patient empowerment slightly increased in the IG, while it slightly decreased in the CG. The self-efficacy remained almost constant in both groups (Supplementary Table 4).

**Supplementary Table 4: Estimated marginal means and 95% confidence intervals for the changes in exploratory endpoints at 3 and 6 months based on the intention-to-treat sample.**

| Endpoint | Analysis | Group | Visit 2 (90d ± 30) | Visit 3 (180d ± 60) |
|---|---|---|---|---|
| **Well-Being** | **CIR** | CG | 0.75 [-0.75, 2.26] | 0.24 [-1.27, 1.75] |
| | **CIR** | IG | 1.33 [-0.13, 2.79] | 1.03 [-0.44, 2.50] |
| **Empowerment** | **CIR** | CG | 0.05 [-0.12, 0.21] | -0.03 [-0.18, 0.13] |
| | **CIR** | IG | 0.14 [-0.01, 0.29] | 0.18 [0.03, 0.34] |
| **Self-Efficacy** | **CIR** | CG | 0.02 [-0.20, 0.25] | -0.04 [-0.27, 0.20] |
| | **CIR** | IG | 0.02 [-0.18, 0.23] | -0.01 [-0.24, 0.21] |
| **Self-Management** | **CIR** | CG | 0.34 [-0.01, 0.70] | 0.11 [-0.24, 0.46] |
| | **CIR** | IG | 0.36 [0.01, 0.71] | 0.44 [0.09, 0.79] |
| **Depression severity** | **CIR** | CG | -0.47 [-1.86, 0.92] | -0.79 [-2.12, 0.55] |
| | **CIR** | IG | -1.39 [-2.69, -0.09] | -1.63 [-2.92, -0.35] |
| **Physical Quality of Life** | **CIR** | CG | 0.60 [-1.44, 2.63] | 1.34 [-0.77, 3.45] |
| | **CIR** | IG | 1.18 [-0.80, 3.15] | 0.51 [-1.44, 2.46] |
| **Psychological Quality of Life** | **CIR** | CG | 1.62 [-1.93, 5.17] | -0.79 [-4.49, 2.90] |
| | **CIR** | IG | 1.06 [-2.29, 4.41] | 0.93 [-2.57, 4.43] |
| | **CIR** | CG | 1.09 [-1.52, 3.70] | 0.01 [-2.62, 2.64] |

| | | | | |
|---|---|---|---|---|
| **Waist Circum-ference (in cm)** | **CIR** | IG | -0.54 [-3.14, 2.06] | -1.24 [-3.85, 1.38] |

*Abbreviations: CG, control group; d, days; CIR; copy increments to reference; IG; intervention group.*

In the ANCOVAs, the main factors of the exploratory endpoints did not yield significant (Supplementary Table 5). The significant results of the respective baseline values, as confounding factors, in all exploratory endpoints, apart from the waist circumference, indicates different outcomes based on the baseline situation. For the physical quality of life, the confounder *gender* was further significant (Supplementary Table 5).

**Supplementary Table 5: Results of the ANCOVA for the exploratory endpoints based on the intention-to-treat sample.**

| Endpoint | Analysis | Effect | *df* | *F-value* | *p-value* |
|---|---|---|---|---|---|
| **Well-Being (WHO-5)** | **CIR** | time | 1, 146 | 0.83 | 0.363 |
| | **CIR** | group | 1, 239 | 0.57 | 0.451 |
| | **CIR** | baseline HbA1c | 1, 218 | 0.41 | 0.524 |
| | **CIR** | baseline well-being | 1, 230 | 16.25 | < .0001*** |
| | **CIR** | gender | 1, 238 | 0.11 | 0.741 |
| | **CIR** | center | 5, 279 | 0.11 | 0.990 |
| | **CIR** | group * time | 6, 279 | 0.11 | 0.995 |
| **Empowerment (DES-SF)** | **CIR** | time | 1, 182 | 1.03 | 0.312 |
| | **CIR** | group | 1, 236 | 1.49 | 0.224 |
| | **CIR** | baseline HbA1c | 1, 176 | 0.36 | 0.550 |
| | **CIR** | baseline DES-SF | 1, 131 | 25.87 | < .0001*** |
| | **CIR** | gender | 1, 226 | 0.61 | 0.435 |
| | **CIR** | center | 5, 271 | 0.74 | 0.593 |

| | | | | | |
|---|---|---|---|---|---|
| | **CIR** | group * time | 6, 274 | 0.89 | 0.505 |
| **Self-Efficacy (ASKU)** | **CIR** | time | 1, 169 | 0.23 | 0.631 |
| | **CIR** | group | 1, 233 | 0.00 | 0.999 |
| | **CIR** | baseline HbA1c | 1, 147 | 3.27 | 0.072 |
| | **CIR** | baseline ASKU | 1, 130 | 43.33 | < .0001*** |
| | **CIR** | gender | 1, 194 | 3.23 | 0.074 |
| | **CIR** | center | 5, 269 | 0.95 | 0.452 |
| | **CIR** | group * time | 6, 273 | 0.80 | 0.571 |
| **Self-Management (SDSCA-G)** | **CIR** | time | 1, 198 | 2.99 | 0.086 |
| | **CIR** | group | 1, 217 | 0.01 | 0.924 |
| | **CIR** | baseline HbA1c | 1, 171 | 1.27 | 0.262 |
| | **CIR** | baseline SDSCA-G | 1, 179 | 22.90 | < .0001*** |
| | **CIR** | gender | 1, 192 | 0.21 | 0.650 |
| | **CIR** | center | 5, 257 | 0.39 | 0.852 |
| | **CIR** | group * time | 6, 260 | 0.82 | 0.552 |
| **Depression severity (PHQ9)** | **CIR** | time | 1, 164 | 0.41 | 0.523 |
| | **CIR** | group | 1, 230 | 1.76 | 0.186 |
| | **CIR** | baseline HbA1c | 1, 207 | 0.05 | 0.828 |
| | **CIR** | baseline PHQ9 | 1, 219 | 25.77 | < .0001*** |
| | **CIR** | gender | 1, 214 | 0.03 | 0.857 |
| | **CIR** | center | 5, 277 | 0.23 | 0.949 |

| | | | | | |
|---|---|---|---|---|---|
| | **CIR** | group * time | 6, 278 | 0.19 | 0.979 |
| **Physical Quality of Life (SF12)** | **CIR** | time | 1, 168 | 0.66 | 0.417 |
| | **CIR** | group | 1, 226 | 0.28 | 0.594 |
| | **CIR** | baseline HbA1c | 1, 192 | 1.67 | 0.198 |
| | **CIR** | baseline SF12 | 1, 210 | 53.56 | < .0001*** |
| | **CIR** | gender | 1, 225 | 6.97 | 0.009** |
| | **CIR** | center | 5, 277 | 0.59 | 0.705 |
| | **CIR** | group * time | 6, 278 | 0.76 | 0.601 |
| **Psychological Quality of Life (SF12)** | **CIR** | time | 1, 142 | 2.69 | 0.103 |
| | **CIR** | group | 1, 242 | 0.10 | 0.752 |
| | **CIR** | baseline HbA1c | 1, 213 | 0.06 | 0.812 |
| | **CIR** | baseline SF12 | 1, 214 | 21.87 | < .0001*** |
| | **CIR** | gender | 1, 201 | 0.47 | 0.494 |
| | **CIR** | center | 5, 278 | 0.40 | 0.850 |
| **Waist circumference (in cm)** | **CIR** | group * time | 6, 278 | 0.66 | 0.681 |
| | **CIR** | time | 1, 150 | 2.89 | 0.091 |
| | **CIR** | group | 1, 222 | 3.00 | 0.085 |
| | **CIR** | baseline waist circumference | 1, 233 | 2.84 | 0.093 |
| | **CIR** | baseline SF12 | 1, 218 | 0.39 | 0.535 |
| | **CIR** | gender | 1, 214 | 0.00 | 0.984 |

| | | | | |
|---|---|---|---|---|
| **CIR** | center | 5, 243 | 0.41 | 0.84 |
| **CIR** | group * time | 6, 244 | 0.38 | 0.891 |

*Significance Codes: 0 '***' 0.001 '**' 0.01 '*' 0.05 '.' 0.1 ' ' 1*

*Abbreviations: ASKU; Short Scale for Measuring General Self-efficacy Beliefs (German: Allgemeine Selbstwirksamkeits Kurzskala); CIR, copy increments to reference; DES-SF, Diabetes Empowerment Scale-Short Form; df, degrees of freedom; PHQ-9, Patient Health Questionnaire; SDSCA-G, Summary of Diabetes Self-Care Activites (German Version); SF-12, Short Form Health Survey; WHO-5, World Health Organization-Five Well-Being Index.*

Overall, the exploratory analyses revealed significant improvement in the IG for self-empowerment, self-management and depression severity (Supplementary Table 6). The group-difference in self-management was trend significant (Supplementary Table 6).

**Supplementary Table 6: Results of the planned contrasts for the exploratory endpoints for the intention-to-treat.**

| Hypothesis | Analysis | Inference | | | | |
|---|---|---|---|---|---|---|
| | | Estimate (95% CI) | t-ratio | df | pFDR | d (95% CI) |
| | | ***Well-Being*** | | | | |
| **IG: T0 > T2** | **CIR** | 1.03 [-0.44, 2.5] | 1.379 | 170 | 0.085. | 0.21 [-0.09, 0.51] |
| **IG > CG** | **CIR** | 0.79 [-0.68, 2.26] | 1.060 | 201 | 0.290 | 0.15 [-0.13, 0.43] |
| | | ***Empowerment*** | | | | |
| **IG: T0 < T2** | **CIR** | 0.18 [0.03, 0.34] | 2.326 | 188 | 0.011* | 0.34 [0.05, 0.63] |
| **IG > CG** | **CIR** | 0.21 [0.06, 0.37] | 2.690 | 236 | 0.008** | 0.35 [0.09, 0.61] |
| | | ***Self-Efficacy*** | | | | |
| **IG: T0 < T2** | **CIR** | -0.01 [-0.24, 0.21] | -0.127 | 241 | 0.551 | -0.02 [-0.27, 0.24] |
| **IG > CG** | **CIR** | 0.02 [-0.21, 0.25] | 0.190 | 289 | 0.849 | 0.02 [-0.21, 0.25] |
| | | ***Self-Management*** | | | | |
| **IG: T0 < T2** | **CIR** | 0.44 [0.09, 0.79] | 2.513 | 183 | 0.006** | 0.37 [0.08, 0.66] |

| | | | | | | |
|---|---|---|---|---|---|---|
| **IG > CG** | **CIR** | 0.33 [0, 0.67] | 1.950 | 228 | 0.053. | 0.26 [0.00, 0.52] |
| | | | ***Depression severity*** | | | |
| **IG: T0 > T2** | **CIR** | -1.63 [-2.92, -0.35] | -2.515 | 165 | 0.006** | -0.39 [-0.7, -0.08] |
| **IG < CG** | **CIR** | -0.85 [-2.16, 0.46] | -1.280 | 192 | 0.203 | -0.18 [-0.47, 0.10] |
| | | | ***Physical Quality of Life*** | | | |
| **IG: T0 < T2** | **CIR** | 0.51 [-1.44, 2.46] | 0.518 | 187 | 0.303 | 0.08 [-0.21, 0.36] |
| **IG > CG** | **CIR** | -0.83 [-2.97, 1.31] | -0.760 | 232 | 0.446 | -0.1 [-0.36, 0.16] |
| | | | ***Psychological Quality of Life*** | | | |
| **IG: T0 < T2** | **CIR** | 0.93 [-2.57, 4.43] | 0.524 | 182 | 0.300 | 0.08 [-0.21, 0.37] |
| **IG > CG** | **CIR** | 1.72 [-1.84, 5.29] | 0.950 | 224 | 0.341 | 0.13 [-0.13, 0.39] |
| | | | ***Waist Circumference (in cm)*** | | | |
| **IG: T0 > T2** | **CIR** | -1.24 [-3.85, 1.38] | -0.935 | 132 | 0.176 | -0.16 [-0.5, 0.18] |
| **IG > CG** | **CIR** | -1.24 [-3.1, 0.62] | -1.320 | 160 | 0.189 | -0.21 [-0.52, 0.10] |

*Significance Codes: 0 ‘***’ 0.001 ‘**’ 0.01 ‘*’ 0.05 ‘.’ 0.1 ‘ ’ 1*

*Abbreviations: CG, control group; CI; Confidence-Interval; CIR, copy increments to reference; d, Cohens’’ d; df, degrees of freedom; IG; intervention group; T0, time point 0 (baseline); T2, time point 2 (6 months after baseline).*

## 6) Results of the User Experience Questionnaire

To assess the user experience with the app, the user experience questionnaire (UEQ) was used at the 6 months visit in the intervention group. In total 56 (73.7%) participants of the IG answered the questionnaire. Participants rated each word pair, describing aspects of the app usability, on a scale from 1 to 7, with 7 showing a strong agreement with the word on the left or the right. For most descriptive word pairs, participants agreed with the positive adjective on the left, indicating a positive evaluation of mebix and its usability (green area in Supplementary Figure 1).

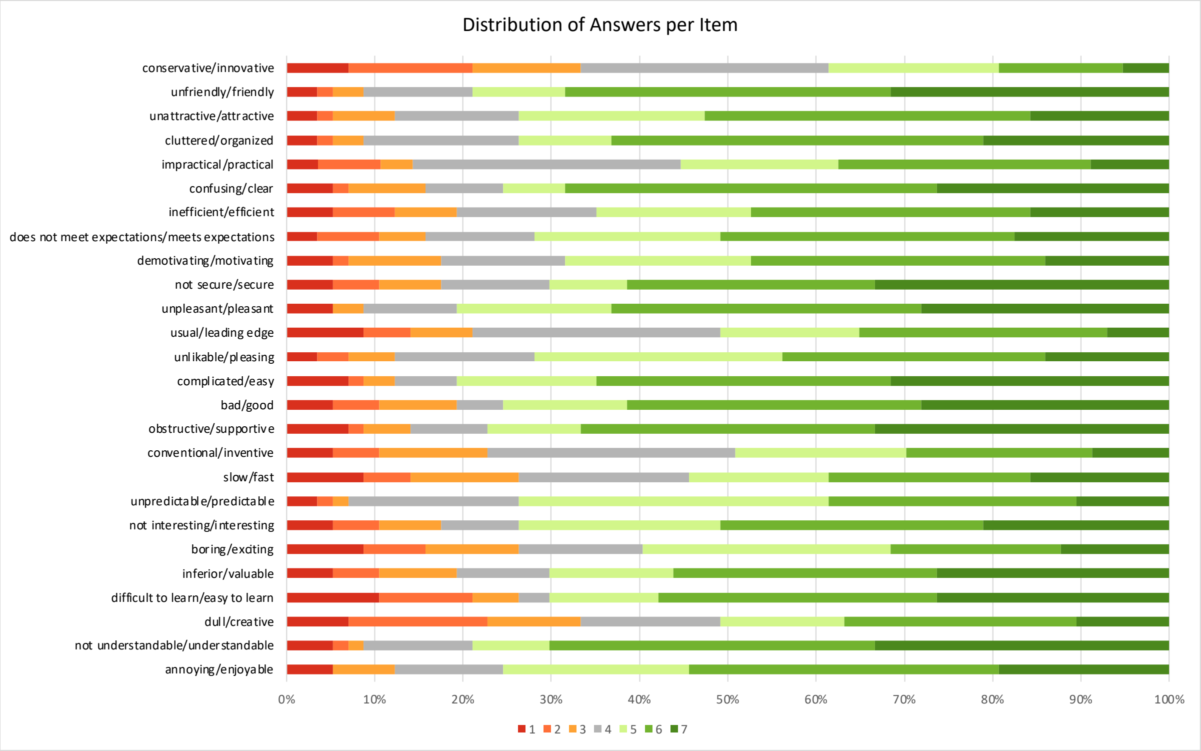


**Supplementary Figure 1: Distribution per Item of the User Experience Questionnaire in the intervention group after 6 months.**

Supplementary Figure 2 shows the results grouped into categories. Categories with a value above 0.8 represents a positive evaluation (while a value below 0.8 representing a negative evaluation). *mebix* received the highest score for *perspicuity* (1.37; variance: 1.97), followed by *attractiveness* (1.33; variance: 1.62), *dependability* (1.24; variance: 1.86), s*timulation* (0.96, variance: 1.94) and *efficiency* (0.92; variance: 0.96). *Novelty* (0.34; variance: 1.35) was rated neutrally.

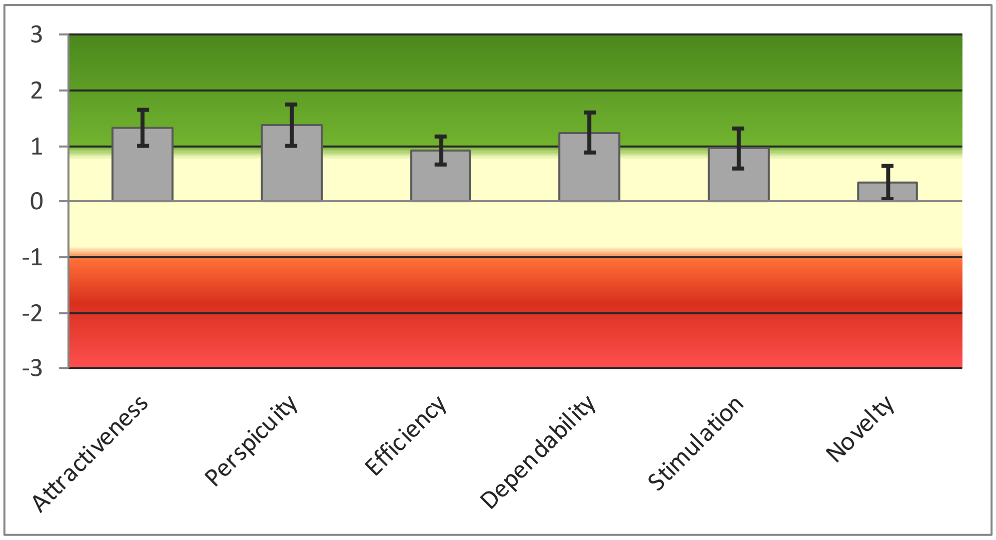


**Supplementary Figure 2: Mean and Variance of the User Experience Categories.**
